# Effect of high-flow nasal therapy on patient-centred outcomes in patients at high risk of postoperative pulmonary complications after cardiac surgery: a study protocol for a multicentre adaptive randomised controlled trial

**DOI:** 10.1186/s13063-022-06180-5

**Published:** 2022-03-28

**Authors:** Melissa Earwaker, Sofia Villar, Julia Fox-Rushby, Melissa Duckworth, Sarah Dawson, Jo Steele, Yi-da Chiu, Edward Litton, Gudrun Kunst, Gavin Murphy, Guillermo Martinez, Vasileios Zochios, Val Brown, Geoff Brown, Andrew Klein

**Affiliations:** 1 Papworth Trials Unit Collaboration, Royal Papworth Hospital, Cambridge, UK; 2 MRC Biostatistics Unit, Cambridge University, Cambridge, UK; 3 King’s College London, London, UK; 4 Papworth Trials Unit, Royal Papworth Hospital, Cambridge, UK; 5 Intensive Care Unit, Fiona Stanley Hospital, Perth, Western Australia Australia; 6 University of Leicester, Leicester, UK; 7 University Hospitals Birmingham, Birmingham, UK; 8 Department of Anaesthesia, Royal Papworth Hospital, Cambridge, UK

**Keywords:** Cardiothoracic surgery, High-flow nasal therapy, Post-operative pulmonary complications, Adaptive design

## Abstract

**Background:**

High-flow nasal therapy is a non-invasive form of respiratory support that delivers low-level, flow dependent positive airway pressure. The device can be better tolerated by patients than alternatives such as continuous positive airway pressure. The primary objective is to determine if prophylactic high-flow nasal therapy after tracheal extubation can result in an increase in the number of days alive and at home within the first 90 days after surgery, when compared with standard oxygen therapy. The co-primary objective is to estimate the incremental cost-effectiveness and cost-utility of high-flow nasal therapy vs standard oxygen therapy at 90 days, from the view-point of the public sector, the health service and patients.

**Methods:**

This is an adaptive, multicentre, international parallel-group, randomised controlled trial with embedded cost-effectiveness analysis comparing the use of high-flow nasal therapy with control in patients at high risk of respiratory complications following cardiac surgery. Participants will be randomised before tracheal extubation and allocated either high-flow nasal therapy or standard oxygen therapy for a minimum of 16 h immediately post extubation. Participants will be followed up until 90 days after surgery. The total sample size needed to detect a 2-day increase in DAH90 with 90% power with an intention to treat analysis is 850 patients. The adaptive design includes an interim sample size re-estimation which will provide protection against deviations from the original sample size assumptions made from the single-centre pilot study and will allow for a maximum sample size increase to 1152 patients.

**Discussion:**

Evidence to support routine use of high-flow nasal therapy will inform the development of effective enhanced recovery care bundles. Reducing complications should reduce length of stay and re-admission to hospital and provide an important focus for cost reduction. However; high-quality studies evaluating the clinical and cost effectiveness of high-flow nasal therapy after cardiothoracic surgery are lacking.

**Trial registration:**

The study has been registered with ISRCTN (ISRCTN14092678, 13/05/2020)

Clinicaltrials.gov Registration Pending

## Administrative information

Note: the numbers in curly brackets in this protocol refer to SPIRIT checklist item numbers. The order of the items has been modified to group similar items (see http://www.equator-network.org/reporting-guidelines/spirit-2013-statement-defining-standard-protocol-items-for-clinical-trials/).

**Table:** 

Title {1}	Effect of High-Flow Nasal Therapy on Patient-Centred Outcomes in Patients at High Risk of Postoperative Pulmonary Complications after Cardiac Surgery: A Multicentre Randomised Controlled Trial
Trial registration {2a and 2b}.	The study has been registered with ISRCTN Trial ID: ISRCTN14092678 Date registered: 13/05/2020 ISRCTN is a primary registry of the WHO ICTRP network and includes all items from the WHO Trial Registration data set
Protocol version {3}	Version 3.0 Dated 22^nd^ September 2021
Funding {4}	For UK: National Institute for Health Research (NIHR Health Technology Assessment). Unique Award Identifier NIHR128351 For Australia: Medical Research Future Fund, Australia (APP2006100) For New Zealand: Green Lane Research and Educational Fund (21/23/4159)
Author details {5a}	Ms Melissa Earwaker, Trial Manager (Papworth Trials Unit Collaboration, Royal Papworth Hospital) Dr Sofia Villar, Lead Statistician (MRC Biostatistics Unit: Cambridge University) Professor Julia Fox-Rushby, Lead Health Economist (King’s College London) Ms Melissa Duckworth, Clinical Project Manager (Papworth Trials Unit Collaboration, Royal Papworth Hospital) Ms Sarah Dawson, Statistician (MRC Biostatistics Unit: Cambridge University) Ms Jo Steele, Senior Clinical Trial Data Manager (Papworth Trials Unit Collaboration, Royal Papworth Hospital) Dr Yi-da Chiu, Statistician (Papworth Trials Unit, Royal Papworth Hospital) Associate Professor Edward Litton (Intensive Care Unit, Fiona Stanley Hospital, Perth, Western Australia) Dr Gudrun Kunst, Clinical Co-Investigator (Kings College London) Professor Gavin Murphy, Clinical Co-Investigator (University of Leicester) Dr Guillermo Martinez, Clinical Co-Investigator (Papworth Trials Unit, Royal Papworth Hospital) Dr Vasileios Zochios, Clinical Co-Investigator (University Hospitals Birmingham) Ms Val Brown, Study Patient and Public Involvement Panel Member Mr Geoff Brown, Study Patient and Public Involvement Panel Member Professor Andrew Klein, Principle Investigator (Department of Anaesthesia, Royal Papworth Hospital)
Name and contact information for the trial sponsor {5b}	Papworth Trials Unit Collaboration Royal Papworth Hospital NHS Foundation Trust, Papworth Road, Cambridge Biomedical Campus, Cambridge, CB2 0AY Tel: 01223 638000 Website; https://royalpapworth.nhs.uk/research-and-development Clinical Trials and Data Management Centre (CTDMC) Curtin School of Population Health, Curtin University Building 400, Room 213 50 Kent Street Bentley, Western Australia 6102
Role of sponsor {5c}	Papworth Trials Unit Collaboration (PTUC), a fully accredited UKCRC Clinical Trials Unit, will oversee the study and provide project management oversight, trial management, data management, statistical and health economic analysis and research governance support as well as input into the overall study design, statistical and health economic design. PTUC also hold overall authority over publications in line with funder. Curtin Clinical Trials and Data Management Unit will provide project management oversight and trial management for Australian sites.

## Introduction

### Background and rationale {6a}

Patients undergoing cardiac surgery are at significant risk of postoperative pulmonary complications (PPC) that may lead to prolonged intensive care unit (ICU) and hospital stay and increased mortality [1]. The incidence of respiratory complications is three to four times higher in patients with intrinsic respiratory disease and lower airway obstruction (including asthma or chronic obstructive pulmonary disease (COPD)), obese patients or current heavy smokers (> 10 pack years) [2]. These patients make up around one quarter of those undergoing cardiac surgery, often developing lower respiratory tract infections with impaired oxygenation/ventilation and require prolonged ventilatory support. They are more likely to require postoperative escalation of respiratory support and readmission ICU during recovery from surgery stay longer in hospital and be re-admitted to hospital after discharge due to ongoing complications [3–5].

High-flow nasal oxygen therapy (HFNT) is increasingly used as a non-invasive form of respiratory support [6]. It delivers low level, flow-dependent positive airway pressure, and is much better tolerated by patients than alternatives such as continuous positive airway pressure (CPAP) or non-invasive ventilation [7]. Patients can talk, eat, drink and walk whilst using HFNT. However, there is equipoise regarding its prophylactic use to prevent postoperative respiratory complications (as opposed to treating complications where good evidence current exists) and effect on important patient-centred outcomes, hence the rationale for this study.

Recent systematic reviews in non-cardiac [8] and cardiothoracic surgery [9] concluded that HFNT could reduce the need for respiratory support and pulmonary complications, and could be safely administered. The most recent meta-analysis published in 2017, examined the efficacy and safety of HFNT after cardiac surgery compared with conventional oxygen therapy and found that post-extubation application of HFNT was associated with a significant reduction in escalation of respiratory support (RR, 0.61; 95% CI, 0.46–0.82; *z* = 3.32, *p* < 0.001) [10]. However, the few studies analysed meant methods used to detect publication bias were underpowered. In addition, definitions, strategies and criteria for escalation of respiratory support in studies included in the meta-analysis were non-specific, making validity of the results questionable. Therefore authors recommended that large-scale randomised controlled trials (RCTs) were needed.

Our group performed a single-centre randomised controlled trial investigating the effect of HFNT on clinically relevant outcomes in cardiac surgical patients with pre-existing lung disease [including COPD or asthma] or a higher risk for pulmonary complications (including obesity (BMI > 35 kg.m^2^), recent respiratory tract infections (in preceding four weeks) or current heavy smoking) [11]. Prophylactic use of HFNT in these cardiac surgical patients at higher risk for pulmonary complications was well tolerated with a compliance of 75% in the treatment arm receiving treatment (defined as a minimum of 16 h of randomised treatment), with 12% crossover from standard oxygen therapy (SOT) to HFNT and 25% crossover from HFNT to SOT. In total, 99% of patients provided outcome data at 90 days. We demonstrated a 2-day reduction in the median length of hospital stay (median (IQR [range]) 7 (6–9 [4–30]) days in the high-flow nasal oxygen group and 9 (7–16 [4–120]) days in the SOT group, *p* = 0.012), a reduction in the geometric mean of length of hospital stay by 29% (95% CI 11-44%, *p* = 0.004) and a reduction in intensive care unit (ICU) re-admission rate (1/49 in the HFNT group vs. 7/45 in the SOT group, *p* = 0.026) [11]. This pilot study provided evidence of feasibility and pilot data to help power and design the larger NOTACS multicentre study.

Hospital and ICU stay are likely to form a large portion of the total cost of patient care and therefore provide an important focus for cost reduction [12]. However; no studies on HFNT in cardiothoracic surgery have yet provided adequate costing. Whilst related economic papers in the wider literature (pre-term infants, ICU patients) appear to support the potential for cost saving, significant caveats are given [13]. The proposed study will provide not only the first primary data on the cost and cost-effectiveness analysis of HFNT for adult cardiac surgery, but it may also be of interest for HFNT after other types of major operations, such as major abdominal and thoracic surgery.

#### Burden of disease

Figures from the National Institute for Cardiovascular Outcomes Research (NICOR) database [14] show that, over the last 7 years, an average of 36,505 patients a year underwent cardiac surgery in the UK. Around 26% of these patients would have fulfilled the study inclusion criteria and qualified as high risk for postoperative pulmonary complications and prolonged hospital stay. This equates to approximately 9500 patients at risk for postoperative pulmonary complications per year in the UK.

#### Why this research is needed now

Enhanced recovery after cardiac surgery is an emerging and important concept in perioperative care, designed to reduce complications, hospital stay and health service and resource use [8, 14]. Evidence to support the routine use of HFNT will inform the development of effective enhanced recovery care bundles. However, before the intervention is recommended for routine NHS use in cardiac surgery patients at high risk of pulmonary complications, the hypothesis that HFNT improves patient-related outcomes and is cost effective in a UK setting needs to be tested.

#### Potential NHS cost savings

Data from the pilot study showed that patients, at high risk of postoperative pulmonary complications receiving prophylactic HFNT stayed on average 2 days less in hospital and ICU re-admission was reduced from 15% to 2% when compared to similar high risk patients receiving SOT [11]. If this pilot data is extrapolated to the eligible UK population, there is the potential to save 19,000 hospital bed days and 1235 re-admissions to ICU each year, each with a median ICU stay of 4 days (the target set by the Getting it Right First Time [GIRFT] cardiothoracic report was 3.2 days) [10]. Such savings in ICU and surgical ward bed days would allow either more patients to be treated within the same cardiac surgery resource in the same number of in-patient beds or alternatively allow a reduction in capacity in NHS cardiac surgery beds, thus freeing up resources to treat other patients (termed ‘notional financial opportunity’ in the GIRFT cardiothoracic surgery report). Using cost data from the GIRFT report [10], this new intervention could potentially achieve an NHS cost saving of £6,935,000 per year in surgical ward bed days (at a cost of £365 per day) and a further saving, from reduced readmission to critical care, of £6,224,000 (£1260/day, median 4 days and 1235 re-admissions) per year.

### Objectives {7}

To determine if prophylactic use of HFNT (for a minimum of 16 h after tracheal extubation) is clinically and cost-effective up to 90 days after surgery, for adult patients undergoing cardiac surgery with cardiopulmonary bypass who are at high risk of postoperative pulmonary complications.

#### Primary outcomes

- To determine if prophylactic HFNT therapy after cardiac surgery in patients at high-risk of developing pulmonary complications results in an increase in DAH90 (days alive and at home in 90 days)
- To estimate the incremental cost-effectiveness and cost-utility of HFNT versus SOT at 90 days

#### Exploratory secondary outcomes

- Incremental cost-effectiveness and cost-utility of HFNT versus SOT at 30 days
- DAH30
- Mortality
- Postoperative pulmonary complications [15]
- ICU re-admission rate
- Total length of ICU stay (days) [16]
- Total length of hospital stay (days)
- Readmission to hospital
- Incidence of stroke
- Incidence of sepsis
- Incidence of myocardial infarction
- Incidence of acute kidney injury
- Oxygenation, as measured by ROX Index (defined as Sp02/FiO_2_ to respiratory rate ratio) [17]
- Patient-reported outcomes (EQ- 5D-5L)
- Patient level of assistance needed with Activities of Daily Living (BARTHEL questionnaire) [18, 19]
- Quality of survival (EQ-5D-5L quality-adjusted life years (QALYs) [20])
- Health service and resource use

#### Trial design {8}

**Fig. 1:**
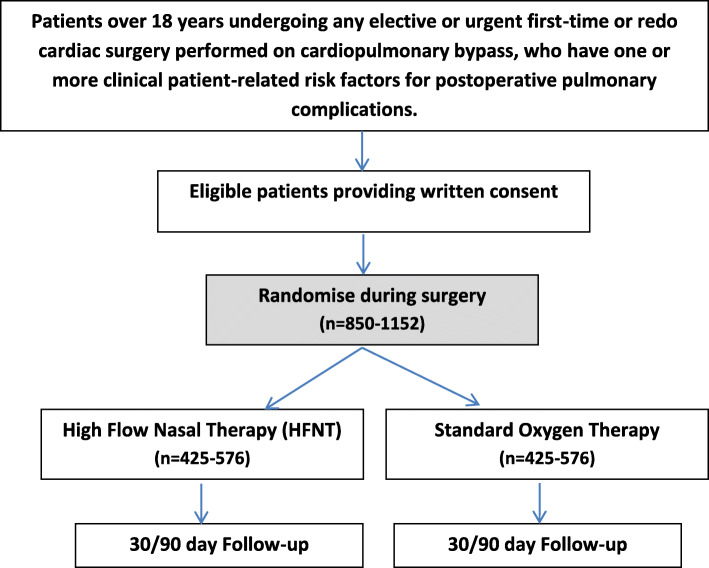
Patient flow diagram

The study is an adaptive, multicentre, parallel group, RCT with embedded cost-effectiveness analysis comparing the use of HFNT, to SOT for a minimum of 16 h after tracheal extubation, in patients at high risk of respiratory complications following cardiac surgery. Patients will be randomly assigned to receive either HFNT or SOT in a 1:1 allocation ratio. The patient flow can be seen in Fig. 1.

## Methods: participants, interventions and outcomes

### Study setting {9}

Patients will be recruited over a period of approximately 4 years from a minimum of 10 cardiothoracic surgery units across the UK, 8 centres in Australia and 1 centre in New Zealand. They will be recruited through elective cardiac surgery pathways as well as urgent admission pathways by members of the study team.

### Eligibility criteria {10}

#### Inclusion criteria

1. Aged 18 years or over
2. Undergoing any elective or urgent first-time or redo cardiac surgery performed on cardiopulmonary bypass
3. Have one or more clinical patient-related risk factor for postoperative pulmonary complications (COPD, asthma, lower respiratory tract infection in last 4 weeks as defined by use of antibiotics, body mass index ≥ 35 kg/m^2^, current (within the last 6 weeks) heavy smokers (> 10 pack years)) [20, 21].
  For the purposes of the study, the following definitions apply:
  Smoking pack years = number of cigarettes smoked per day × number of years smoked
  Asthma is a disease characterized by recurrent attacks of breathlessness and wheezing, and patients will have been prescribed medication by inhalers or nebulisers (either bronchodilators or steroids).
  Chronic obstructive pulmonary disease (COPD) is an umbrella term used to describe chronic lung diseases that cause limitations in lung airflow. The more familiar terms ‘chronic bronchitis’ and ‘emphysema’ are no longer used but are now included within the COPD diagnosis. The most common symptoms of COPD are breathlessness, or a ‘need for air’, excessive sputum production, and a chronic cough. Patients suitable for the NOTACS study will have been prescribed medication by inhalers or nebulisers (either bronchodilators or steroids).

#### Exclusion criteria

1. Requiring home oxygen therapy
2. Planned deep hypothermic circulatory arrest
3. Contraindication to HFNT, e.g. nasal septal defect
4. Requirement for home respiratory support (including CPAP, BiPAP)
5. Requiring emergency cardiac surgery defined as surgery required within 24 h of the decision to operate

- Patients not fluent in English and an interpreting service is likely to be unavailable for some or all of the study duration

Patients meeting all eligibility criteria will be given or sent a patient information sheet and an invitation letter and then either approached by telephone or face to face. Patient requiring urgent surgery after admission to hospital will be given a patient information sheet during admission and then approached face to face to participate in the study prior to surgery.

#### Coronavirus advice

Elective cardiac surgery patients will be, by definition, COVID-19 negative. The NOTACS study participant population is limited to elective and urgent cardiac surgery patients, both of whom undergo COVID-19 testing prior to surgery. Patients requiring emergency cardiac surgery are excluded from the protocol. Therefore, potential participants are unlikely to be positive for COVID-19. NOTACS study recommends sites to follow their local patient pathways and procedures in regards to COVID-19 testing and personal protective equipment (PPE).

### Who will take informed consent? {26a}

Written informed consent will be obtained by a member of the study team, delegated by the principal investigator. This will occur after eligibility has been confirmed, the patient has read the participant information sheet and all questions from the patient have been answered.

### Additional consent provisions for collection and use of participant data and biological specimens {26b}

The informed consent gained from participants for this study also includes the transfer of personal details to the central trials unit at Royal Papworth Hospital NHS Foundation Trust (for UK recruited patients), Curtin University Clinical Trials and Data Management Unit (for Australian recruited patients) and a blinded team based at Auckland City Hospital (for New Zealand recruited patients), via an encrypted database, for the completion of blinded follow-ups. Consent also allows the central trials units to request and hold additional information acquired from the participants’ GP, other hospitals or healthcare providers regarding any hospital admissions after discharge. This additional information will be held confidentially and will be destroyed at the end of the study. Additionally, participants will be asked to provide consent for this information to be collected and copies of discharge summaries to be transferred confidentially from their recruiting hospital.

### Interventions

#### Explanation for the choice of comparators {6b}

HFNT is increasingly used as a non-invasive form of respiratory support [6]. The device provides warmed humidified oxygen mixed with air and has been shown to assist breathing and improve patient recovery. It reduces work of breathing by improving respiratory mechanics and generation of low level positive end-expiratory pressure (PEEP) [15–17] and also inhibits bronchomotor response and reduces bronchospasm [18, 19]. The generation of PEEP level of 3–5 cmH_2_O may also reduce postoperative atelectasis [5, 18, 19]. This reduces postoperative hypoxaemia, hypercapnia and pulmonary morbidity, which means complications due to acute exacerbation of chronic lung conditions are potentially reduced.

HFNT will be compared against SOT of dry oxygen via a facemask or nasal prongs, which is currently standard practice.

#### Intervention description {11a}

As a pragmatic study, perioperative management (anaesthetic technique, surgical procedure, intra-operative mechanical ventilation strategy, and postoperative invasive mechanical ventilation weaning strategy) will not be affected by patients’ participation in the study and will be conducted according to usual local practice.

Randomisation will be performed after initiation of cardiac surgery and prior to extubation. Patients will be randomly assigned to receive either HFNT or SOT using an online tool (provided by Sealed Envelope). Randomisation will be stratified by centre.

After cardiac surgery, patients will be transferred sedated and with their trachea intubated to the post-surgical recovery area. This may be an ICU, high dependency unit (HDU) or specific recovery unit as per local practice. Once patients fulfil the local hospital criteria for extubation (for example minimal bleeding via chest drains; temperature > 36 °C; stable cardiovascular status; neuromuscular blockade worn off or reversed; sedation stopped; patients responsive to command and successful spontaneous breathing trial (defined as oxygen saturations (SpO_2_) > 93% with inspired oxygen less than or equal to 60% and adequate spontaneous effort), they will then be extubated following the trial extubation protocol (see Table 1) and will receive either HFNT or SOT for a minimum of 16 h according to their randomised allocation. Patients will be transferred to the surgical ward as per local practice and will be assessed at least every 24 h as per local practice—if SpO_2_ > 93% on air and respiratory rate (RR) < 20 breathes per minute, then HFNT or SOT will be discontinued. If SpO_2_ < 93% or RR < 20, then HFNT or SOT will be continued for a further 24 h then the patient will be re-assessed every 24 h. If a patient deteriorates during HFNT or SOT, then the agreed Trial Escalation of Respiratory Therapy Protocol will be applied (please refer to the “22” section).

**Table 1:** Trial extubation protocol

*Mechanical ventilation and tracheal extubation after cardiac surgery* Patients’ lungs will typically be mechanically ventilated with FiO2 40–60%, PEEP 5–10 cm H_2_O, tidal volume (TV) 5–8 ml/kg ideal body weight and RR 10-20 breaths/min to achieve PaO_2_ > 8 kPa, PaCO_2_ 4–6 KPa and peak pressure < 30 cms H_2_O. If failing to achieve these parameters, ventilator settings may be adjusted, and medical team consulted for advice. The aim is to wean the patient from mandatory ventilation and switch to spontaneous breathing using pressure support (PS)/continuous positive airway pressure (CPAP) as soon as possible. Once the patient is awake and breathing spontaneously, test the patient’s ability to breathe whilst receiving minimal ventilator support via a spontaneous breathing trial (SBT) using PS/CPAP, FiO_2_ < 40%, PS 5–10 cm H_2_O and PEEP 5–10 cm H_2_O. If after spontaneous breathing trial, the patient remains stable, there are no signs of respiratory distress and oxygen saturations > 93% with inspired oxygen less than or equal to 60%, the patient’s trachea should be extubated. If not ready for extubation, then re-assess and repeat SBT as appropriate. If patient continually fails SBT, then discuss with medical team. To proceed to extubation patients should be: - Able to follow commands - Able to protect own airway - Have adequate strength (e.g. lift head off pillow) - Have adequate respiratory effort - Haemodynamically stable - Bleeding within expected limits (as per local protocol) - Adequately reversed (neuromuscular blockade) After extubation, immediately apply high-flow nasal therapy or standard oxygen depending on group allocation.	
*High-flow nasal therapy* High-flow nasal therapy equipment and disposables should be prepared in advance and checked whilst patient’s lungs still being mechanically ventilated Start at 40% inspired O_2_ and flow 30 l/min then up to 50 l/min over 5–10 min. Monitor saturations and RR and arterial gases after 15 min then as per local policy. If saturations < 93%, then increase FiO_2_ as per respiratory escalation protocol.	
*Standard oxygen therapy* Start 30–40% inspired O2 and flow 2–6 l/min via nasal prongs or non-rebreathing mask (not humidified and not heated). Monitor saturations and RR and arterial gases after 15 min then as per local policy. If saturations < 93%, then increase FiO_2_ as per respiratory escalation protocol.	
*Ideal body weight is the weight corresponding to an ideal body mass index of 22 kg/m^2^ Men IBW = (height in metres)2 × 22 Women, IBW = IBW = (height in metres − 10 cm)2 × 22	

#### Criteria for discontinuing or modifying allocated interventions {11b}

All patients on oxygen therapy (HFNT or SOT) should have regular pulse oximetry measurements. The frequency of oximetry measurements will depend on the stability of the patient. Critically ill patients should have their oxygen saturations monitored continuously and recorded every few minutes whereas patients with mild breathlessness will need less frequent monitoring. Oxygen therapy should be increased if the SpO_2_ is < 93% and decreased if the SpO_2_ is > 95% (and eventually discontinued as the patient recovers). Any sudden fall in oxygen saturation should lead to clinical evaluation of the patient and in most cases, measurement of blood gases after medical review. All hemodynamically unstable and critically ill patients should be given 100% oxygen (15 l/min reservoir mask) whilst awaiting immediate medical review. Full protocol for escalation of respiratory therapy protocol can be seen in Table 2.

**Table 2:** Trial escalation of respiratory therapy protocol

All patients on oxygen therapy (HFNT or standard therapy) should have regular pulse oximetry measurements. The frequency of oximetry measurements will depend on the stability of the patient. Critically ill patients should have their oxygen saturations monitored continuously and recorded every few minutes whereas patients with mild breathlessness will need less frequent monitoring. Oxygen therapy should be increased if the saturation is < 93% and decreased if the saturation is > 95% (and eventually discontinued as the patient recovers). Any sudden fall in oxygen saturation should lead to clinical evaluation of the patient and in most cases, measurement of blood gases. All peri-arrest and critically ill patients should be given 100% oxygen (15 l/min reservoir mask) whilst awaiting immediate medical review. Escalation of respiratory therapy may be indicated if: - Saturations < 93% - RR > 20 breaths/min - PaCO_2_ > 7 kPa	
Plan A Assess patient, consider chest x-ray Increase FiO2 in increments of 10% up to a maximum of 60%. If patient is receiving high-flow nasal therapy, consider increasing flow up to max 60 l/min	
Plan B Assess patient, consider chest X-ray and arterial blood gas Consider transfer to level 2 or level 3 care environment (HDU or ICU) Increase FiO_2_ in increments of 10% up to a maximum of 100% Consider CPAP (mask or nasal mask or hood), start at 5 cm H_2_O Consider non-invasive ventilation (NIV) or BiPAP	
Plan C Assess patient, consider chest X-ray and arterial blood gas Consider invasive mechanical ventilation (requires tracheal intubation)	
Clinicians can move between plans A, B and C depending on the patient’s condition and not necessarily in that order.	

#### Strategies to improve adherence to interventions {11c}

All study staff, both clinical and non-clinical, will receive protocol and device training (if required) before being entered onto the delegation and training logs to ensure protocol adherence. Clinically, protocols will be laminated and paced by the study team at each participant’s bedside when the patient is admitted to the post-surgery care area and the randomised therapy will be set up with the bedside nurse so that it is ready for when the patient’s trachea is extubated. This is to ensure all clinical members of staff are aware of study protocols.

#### Relevant concomitant care permitted or prohibited during the trial {11d}

Routine standard of care is encouraged during all aspects of the study. However, patients are to receive at least 16 h of randomised therapy. The study asks for the protocol to be followed for tracheal extubation at which randomised therapy is initiated and also for escalation of care if there is any deterioration in respiratory function after tracheal extubation.

#### Provisions for post-trial care {30}

Study participants will complete the study follow-ups up to 90 days post-surgery, with serious adverse events being recorded up to this time point. At the 90-day follow-up, participation within the study will be complete for all participants.

### Outcomes {12}

#### Definition and measurement of primary outcome of DAH90:

‘Days alive and at home’ (DAH) after surgery [10, 22] is a validated patient-centred outcome metric that is highly sensitive to changes in surgical risk and impact of complications and has prognostic importance. The choice of primary endpoint (DAH90) is aimed at mitigating potential sources of bias due to the unblinded nature of the study. Following the approach of Myles et al. [22], patients who died within 90 days of surgery will be assigned a zero DAH score irrespective of whether they spent any time at home during the 90 day follow-up period.

Home will be defined as a patient’s usual abode. Home will exclude any nursing facility (rehabilitation centre or nursing home) unless this was the patient’s residence at admission and they return ‘home’ with no increase in level of care. Any hospital readmissions within 90 days of surgery are subtracted from the total. DAH90 will be calculated using mortality and hospitalisation data from the date of randomisation, which is the day of surgery (day 0).

#### Definition and measurement of incremental cost effectiveness for 30 days and 90 days

The incremental cost-effectiveness ratio (ICER) reflects the difference in costs between two interventions divided by the difference in effects (such as QALYs). The statistic is interpreted in relation to threshold values for the willingness to pay for QALYs. By presenting this statistic in association with its uncertainty and threshold values, cost-effectiveness acceptability curves can be mapped to show the probability that an intervention is cost-effective at different willingness to pay for QALY values. A health economic analysis to estimate the incremental cost-effectiveness and cost-utility of HFNT versus SOT at 30 and 90 days will be undertaken.

### Definition of exploratory secondary outcomes

#### Stroke

Stroke is defined as an episode of acute neurological dysfunction due to infarction; ischemia; intracerebral haemorrhage; subarachnoid haemorrhage; cerebral venous thrombosis or unknown cause, persisting ≥ 24 h or until death [23].

#### Sepsis

Sepsis is defined as a life-threatening organ dysfunction caused by a dysregulated host response to infection. Organ dysfunction is identified as an acute change in total sequential organ failure assessment (SOFA) score ≥ 2 points consequent to the infection [24].

#### Acute kidney injury (AKI)

Acute kidney injury (AKI) is defined and staged according to the kidney disease improving global outcomes (KDIGO) criteria [25], reporting all stages.

#### Myocardial infarction

Myocardial infarction is the detection of a rise of cardiac troponin values with at least one value above the 99th percentile along with symptoms of acute myocardial ischaemia; or new ischaemic ECG changes; or development of pathological Q waves; or imaging evidence of new loss of viable myocardium or new regional wall motion abnormality in a pattern consistent with an ischaemic aetiology; or identification of a coronary thrombus by angiography including intracoronary imaging or by autopsy [26].

#### Measurement of exploratory secondary outcomes

Clinical outcomes, resource and health service use will be completed by research nurses on a bespoke case report form (CRF) using patient records to collect inpatient stay data. Patient-reported outcomes will be collected using the EQ-5D-5L [18] and patient level of assistance needed with Activities of Daily Living with be collected using the BARTHEL [19]. Both of which will be completed by the patient.

Costs borne by patients and families (and health service use after the first admission) will be collected using bespoke questionnaires, including the following: out-of-pocket expenses for residential care and assisted living care, care-related expenditure on travel, equipment and prescriptions and days of unpaid family care (specifying whether this includes days off work). These will be completed at baseline, discharge, 30 and 90 days post-surgery.

#### Participant timeline {13}

The participant timeline is shown in Table 3.

**Table 3:** Schedule Of events

Visit number	Visit 1 Screening	Visit 2 Baseline	Visit 3 Randomisation	Visit 4 Discharge	Visit 5 30 days (+ 7 days) Post-op	Visit 6 90 days (+ 7 days) Post-op
Time interval of visit	Prior to surgical admission (or after admission if in-house urgent)	Surgery admission	During or after surgery and pre-extubation	Day of discharge	30 days (+ 7 days) post-op	90 days (+ 7 days) post-op
Activity						
Inclusion/exclusion criteria	**X**					
Informed consent		**X**				
Demographics		**X**				
Past medical history		**X**				
EuroSCORE II and ARISCAT risk assessments		**X**				
EQ-5D-5L and BARTHEL questionnaires		**X**		**X**	**X**	**X**
Participant and family resource use questionnaire		**X**		**X**	**X**	**X**
Adverse and serious adverse events assessed (from the point of extubation			**X-------------------------------------------------------------X**			
Inpatient medication log (to start from the point of extubation)			**X-----------------------X**			
Inpatient location log (to start from the point of extubation)			**X-----------------------X**			
Inpatient oxygen therapy log (to start from the point of extubation)			**X-----------------------X**			
Participant location and medication diary				**X-------------------------------------X**		
Randomisation/initiation of HFNT or standard oxygen therapy			**X**			
ROX Index			**X-----------------------X**			
Record of respiratory support escalation			**X-----------------------X**			
Record of post-operative complications			**X-----------------------X**			
Record of intensive care length of stay and re-admissions				**X**		
Record of hospital discharge destination				**X**		
Record of hospital length of stay				**X**		

#### Sample size {14}

Results from the pilot study [11] and information provided by collaborative hospitals were used to derive the required sample size for the NOTACS study. The sample size calculation relied on several parameters that were provided from the pilot study and may differ between sites in the multicentre design; because of this uncertainty, the NOTACS study includes an interim sample size re-estimation, a type of adaptive design. This will provide protection against important deviations from the original sample size assumptions. The minimum target sample size (based on original assumptions) is 850 randomised participants. The adaptive design will allow for a maximum sample size increase to 1152 patients.

The primary endpoint (DAH90) typically has a left-skewed bimodal distribution with a small spike at 0 due to deaths. The required sample size was obtained by simulations (100,000 replicates) by first generating length of stay (LOS) using a lognormal distribution and derived through a pooled weighted average. The variability was calibrated to SD = 12.85 in the control arm and SD = 3.20 in the treatment arm. The median LOS in the control arm was set to 8 days. We assumed a 3% death rate, and LOS was truncated at 90 days (the maximum for our follow-up period). Finally, DAH90 was computed as 90 minus LOS.

A total sample size of *n* = 310 has 90% power to detect an increase of 2 days in the median DAH90 using the Mann-Whitney-Wilcoxon test for the analysis. After adjustment for 12% crossover from SOT to HFNT and 25% crossover from HFNT to SOT as well as an extra 5% loss to follow-up (equally distributed amongst arms), the total sample size needed to detect a 2-day increase with 90% power with an intention to treat analysis is 850 patients.

#### Recruitment {15}

In addition to an internal pilot phase provided by the sample size re-estimation included in the adaptive design, an internal three-month pilot phase in each study centre to enhance the efficiency and internal validity of the main study will be used. This will focus largely on recruitment, randomisation, intervention and follow-up assessments. The potential timings of reaching the specific sample sizes were estimated via the initial predictive model of patient recruitment numbers [27]; however, it is anticipated that the COVID-19 pandemic will impact on the rate of recruitment. Furthermore, there will be at least two points in the study for trial statisticians to aid further discussion on recruitment performance with predictions: at 10 months after the recruitment commences to predict whether the initial interim is attainable and again, immediately after the interim analysis to foresee whether the revisited target is achievable in the time.

The recruitment monitoring reference has the primary basis on the lower bound of the predicted recruited number. The study team will monitor recruitment performance and prepare for necessary actions. There will be a formal assessment of recruitment at month 15 or after 168 patients have been enrolled. The Trial Steering Committee will assess recruitment against monthly targets to consider whether further sites should be recruited.

It is expected that each centre will recruit a minimum number of patients per month over each 3 month period to get a ‘green light’ to continue in the study. If this minimum target is not met, the site will go ‘amber’ and be given another 3 months to meet minimum recruitment, then if this is not met the site will go ‘red’ and if recruitment cannot be improved in a further 3 months the site will be removed from the study and another site activated. The Sponsor, CTU and Local CRN Research Delivery teams will give sites labelled amber or red all available assistance to improve recruitment.

### Assignment of interventions: allocation

#### Sequence generation {16a}

Patients will be randomly assigned to receive either HFNT or SOT in a 1:1 allocation ratio using an online tool (provided by Sealed Envelope). Randomisation will be stratified by centre. Random permuted blocks within strata will be used to reduce predictability of the randomisation sequence.

#### Concealment mechanism {16b}

An online computer-generated system will conceal the randomisation sequence until planned initiation of the study intervention.

#### Implementation {16c}

The allocation sequence will be computer generated by Sealed Envelope. A member of the study site team will take valid informed consent and then enrol each participant in the study. Once this has been complete, the study site staff shall randomise the patient using Sealed Envelope. As all study site staff and participant are unblinded, a member of the study site staff shall set-up the allocated treatment for the patient.

### Assignment of interventions: blinding

#### Who will be blinded {17a}

Due to the nature of the intervention, it is not possible for patients to be blinded to their randomised treatment allocation. We acknowledge that this has the potential to cause bias, particularly for patient-reported outcomes. However, the choice of primary endpoint (DAH90) being collected using blinded central study team members and a blinded database is aimed at mitigating potential sources of bias due to the unblinded nature of the study.

We have taken the following steps to mitigate detection, procedural and post randomisation bias. Patients will receive the study intervention (HFNT) or SOT immediately after tracheal extubation on the intensive care unit/surgical recovery ward. We have defined a clear study-specific policy for escalation of ventilation support, which includes objective cut points and protocols for oxygen therapy, physiotherapy and other respiratory support that will be used to guide transfer between levels of care. We have mapped out the pathway and specified mild and major non-adherence in an objective way. These deviations will be recorded and reviewed by the Data Monitoring Committee.

#### Procedure for unblinding if needed {17b}

All members of the study site staff and participant are unblinded and therefore will not need emergency unblinding procedures in place. However, if a member of the blinded central team completing follow-ups has need for a participant to be unblinded, a sponsor representative with admin access to Sealed Envelope has the ability to reveal treatment allocation.

## Data collection and management

### Plans for assessment and collection of outcomes {18a}

#### Baseline

Baseline data will be collected following consent including age, sex, ethnicity, skin tone, residential status, past medical history, EuroSCORE II (which predicts mortality at 30 days after surgery) and ARISCAT score (which predicts the risk of in hospital pulmonary complications after surgery, including respiratory failure), quality of life (EQ-5D-5L), activities of daily living (BARTHEL) and health service and resource use questionnaires including antibiotic, cardiac and respiratory medication use.

#### Primary outcome data collection (DAH90)

Data on the primary outcome (DAH90) will be collected using patient diaries up to 90 days postoperatively. Patients will be asked to record every time they change living location and the date they moved. Locations will be pre-coded and defined as: home, hospital, residential home, nursing care, relative’s home or other. Missing data will be followed up by calling the patient’s GP surgery and using hospital discharge summaries for dates of change of living location. Further information regarding death or any additional hospital admission will be collected from the discharge summary and cross-referenced with GP records.

#### Exploratory secondary outcomes

Patient-reported outcomes, patient level of assistance needed and health service and resource use questionnaires will be collected pre-operatively, at discharge from hospital and at 30 and 90 days (+ 7 days) after surgery.

#### EQ-5D-5L

The EQ-5D-5L descriptive system comprises five dimensions: mobility, self-care, usual activities, pain/discomfort and anxiety/depression [23]. Each dimension has 5 ‘problem’ levels: none, slight, moderate, severe and unable to complete. Each dimensions and level are combined into a 5-digit number that describes the patient’s health state [28], e.g. 11111 refers to no problems in any dimension, and 33333 means the patient has moderate problems in each dimension. The cross-walk function from the EQ-5D-3L [29] will be used to value change in health status unless a new valuation set is approved by NICE.

#### BARTHEL activities of daily living

The Barthel Index covers ten domains: feeding, bathing, grooming, dressing, bowel, bladder, toilet, transfers, mobility and stairs. Performance on these domains is rated by level of assistance required.

#### Health service and resource use

Bespoke health service and resource use logs will be completed by the research team using patient records to collect inpatient stay data, e.g. surgery completed, time in theatre and ICU by hours (including returns), types and numbers of tests, procedures, medications, types and treatment for complications, post index-hospital discharge care (days in any hospital, residential care by type), A&E and OPD visits and use of primary care services (e.g.GP/nurse/physio visits, home visits). Oxygen therapy use will be logged by the research team and will include details such as method of oxygen delivery, settings, i.e. number of litres/min and date and time method was initiated and stopped. Research staff will also log antibiotic, cardiac and respiratory medication use as well as in-hospital patient location to include length of stay in each location within hospital, e.g. ITU, HDU and ward setting. Bespoke questionnaires on patient health service and resource us and costs borne by patients and families will include the following: out-of-pocket expenses for residential care and assisted living care, care-related expenditure on travel, equipment and prescriptions and days of unpaid family care (specifying whether this includes days off work). A patient-recorded diary will record changes to daily medications for antibiotic, cardiac and respiratory disease from discharge to 90 days.

#### ROX Index

Data to calculate ROX Index will be collected at 2, 6, 12, 24 and 48 h post extubation. ROX Index = SpO_2_/FiO_2_ to respiratory rate ratio

#### Plans to promote participant retention and complete follow-up {18b}

At consent, participants will be given the option to complete the study follow-ups via a telephone call with the central trials team or electronically online. In order to promote retention, all patients (including those who have chosen online follow-up) will be called by the central trials team at 7 days post discharge to resolve any problems that have arisen in completion of the paper participant location and medication diary. All online follow-up participants will also receive an email and text alert alerting them that a follow-up is due. If a participant does not complete the follow-up online within a set amount of time, the patient will be moved to telephone follow-ups and a member of the central trials team will call the participant. The flow diagram in the “35” section shows time frames. At time of consent, participants consent to a member of the central trials team contacting their GP in the event that contact with a participant has not been successful.

#### Data management {19}

The Papworth Trials Unit Collaboration (PTUC) Data Management team will provide data management oversight for the study and will coordinate with the Statistical and Health Economics teams to ensure that all study data is ready for analysis. Data will be kept on to a bespoke data management database system, OpenClinica, with blinded and unblinded access.

**Fig. 2:**
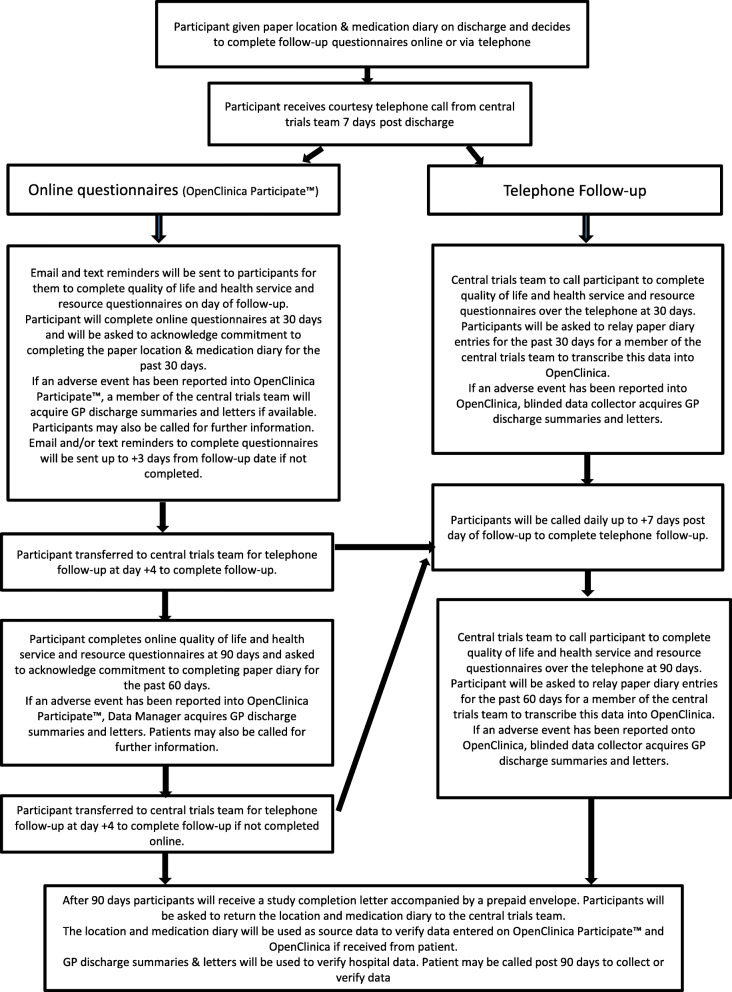
Patient follow-up flow diagram

Following PPI feedback from the pilot study, patients will also be given the opportunity to complete study follow-ups online, with text and email reminders when these are due. The central clinical trials unit staff will ensure online forms have been completed using the escalation process (see Fig. 2). Patients will be telephoned if online forms have not been completed within 4 days of the due date.

#### Source documentation

The investigator and clinical research team must maintain source documents (patient’s medical record) for each patient in the study, consisting of all demographic and medical information.

#### Errors and corrections

A robust audit trail within OpenClinica tracks all changes to the data and retains a history for each variable, including old and new value, date and time of the change and which user created it.

#### Confidentiality {27}

All investigators and study site staff must comply with the requirements all applicable data and privacy laws relevant to the jurisdiction with regard to the collection, storage, processing and disclosure of personal information. All data used in the formulation of reports to investigators, the sponsor, funder or ethics will only contain anonymised data. Patient identifiable information will be stored until the end of the study as per relevant jurisdictions, e.g.15 years in the UK.

#### Plans for collection, laboratory evaluation and storage of biological specimens for genetic or molecular analysis in this trial/future use {33}

This section is not applicable for this study. No collection, laboratory evaluation and/or storage of biological specimens for genetic or molecular analysis are included in this study. All blood result data collected from patients are acquired from routine blood tests.

## Statistical methods

### Statistical methods for primary and secondary outcomes {20a}

#### Statistical analysis

The primary outcome of days alive and at home up to 90 days (DAH90) will be analysed at the end of the study using the Mann-Whitney-Wilcoxon test to account for a skewed sampling distribution. Contrasts for the primary outcome will be used to evaluate the difference in the median DAH90 between the two treatment arms at a 5% significant level. 95% confidence intervals giving a range of plausible effects will be reported.

The primary analysis will be on the basis of intention-to-treat (ITT). The effects of adherence, attrition and likely sources of bias on the primary effect estimate will be evaluated using per protocol, safety and sensitivity analyses. In particular, we will perform a sensitivity analysis for the primary end-point to assess the impact of assigning a DAH score of zero to patients that die at any time within the 90 day follow-up period by relaxing this rule and replacing these zero values by the observed DAH value for these patients.

It is expected that the secondary outcome of days at home up to 30 days (DAH30) will have similar distributional characteristics to that of DAH90. Hence, the Mann-Whitney-Wilcoxon test will also be used to evaluate whether there is a statistically significant difference in the median DAH30 between the two treatment arms.

The statistical analysis will be reported according to CONSORT extension guidelines for adaptive trials [28].

#### Health economics analysis

The base case analysis will be a within-trial, intention-to-treat comparison of the difference in costs falling on the NHS and personal social services with the difference in quality-adjusted life years.

The economic evaluation focuses most data collection on the initial in-patient stay, followed by costing of health and social care service use in the follow-up period. This ensures the detail for expected (e.g. LOS and ICU use) and any unexpected change (e.g. treatment of complications) from the surgical stay is collected and any implications for shifting care to other hospitals/residential care or to patients is captured in broader detail.

The base case economic evaluation will adopt an NHS and personal services view point, with a public sector and patient/family viewpoints included in sensitivity analysis. Intervention costs including set up (e.g. training), initial inpatient care (e.g. length of stay in ward/theatre/ICU including all readmissions, oxygen use by type, treatment of complications, procedures, tests, medication) and follow-up care costs (e.g. readmission to hospital, use of A&E services, appointments and home visits for primary care, use of other community care services, days in residential care by type, medication) to 90 days will be compared with the usual care control. Following discussion with PPI representatives, patient cost data will focus on their largest elements: out-of-pocket expenses for residential care and assisted living, plus days of unpaid caring by family members. Health service and resource use data will be collected from patients and routine sources. Unit costs will be valued using national costs [27], where available, and literature or local costs where not. Outcomes to be used in the economic analysis will include the primary outcome DHA90 and EQ5D5L QALYs with values taken from the ‘cross-walk’ algorithm [29] unless the recent NICE statement [28] is amended.

The results will be provided as total and incremental costs and effects and cost profiles by arm, incremental cost-effectiveness ratios, cost-effectiveness acceptability curves and net-benefit regression, in accordance with good practice guidance and recommendations by NICE [23, 30, 31]. Regression-based analyses of costs and outcomes will account for missingness, censoring, skewness and correlation between costs and outcomes. The effect of baseline characteristics (e.g. EuroSCORE II, ARISCAT score, gender, age, baseline quality of life, residential status and baseline health service use) and any imbalance in covariates on costs and outcomes will be evaluated. Bootstrapping will be used to reflect uncertainty in the incremental cost-effectiveness ratios and correlations between costs/effects. A range of deterministic, scenario and probabilistic sensitivity analyses will investigate the impact of different assumptions (e.g. for valuation), data and approaches (e.g. view point, methods for dealing with missingness) on conclusions. Sensitivity analyses will include viewpoint, alternative methods for dealing with missingness, and any assumptions needed for valuation.

#### Interim analyses {21b}

The assumptions used for the original sample size calculation were based on pilot data [11] and data provided by the largest participating centres. As NOTACS is a multicentre study, using a different primary endpoint to the pilot data, we found that the sample size calculation was very sensitive to the standard deviation, level of treatment switches and loss to follow-up assumed. NOTACS has been designed as an adaptive trial with an interim sample size re-estimation planned after 300 patients complete 90 days post-randomisation follow-up. At the interim sample size re-estimation, we will use the data accumulated so far to re-estimate a number of ‘nuisance’ parameters including standard deviation, treatment switch rate, drop-out rate and death rate.

Treatment efficacy will not be assessed at the interim analysis. Sensitivity analysis will also evaluate the impact of accounting for all days alive and at home (i.e. even if patients die before 90 days) on sample size estimation. This will facilitate discussion of the impact of this assumption on the final estimation of DAH90 and QALYs. These analyses will inform how final analyses for the effectiveness and health economics can be aligned in terms of the primary endpoint definition and used to better address the co-primary questions.

After the interim analysis, the sample size of the study will be updated with a maximum increase up to 1152 patients. There are several possible outcomes from the sample size re-estimation which are summarised in Table 4.

**Table 4:** Recommended sample size from interim sample size re-estimation and course of action

Recommended sample size from interim sample size re-estimation	Course of action
≤ 850	Continue recruitment to 850
851–1152	Continue recruitment to the new recommended sample size
> 1152	Continue recruitment to 1152

The sample size re-estimation will be done using an independent statistician to allow the trial statisticians to remain blinded, in order to preserve the type 1 error rate at 5%. This sample size adaptation may prevent an underpowered study if moderate deviations from the assumptions made for the initial sample size calculation are observed.

#### Methods for additional analyses (e.g. subgroup analyses) {20b}

The statistical analyses will be documented in detail in a statistical analysis plan. Secondary analyses will be performed to explore the secondary outcomes outlined in the “7” section. Sensitivity analyses will be performed in order to evaluate the robustness of the primary analysis, including per-protocol analysis and assessment of missing data.

#### Methods in analysis to handle protocol non-adherence and any statistical methods to handle missing data {20c}

‘Intention to treat’ and ‘per protocol’ analyses will be reported and the extent of bias on the estimates will be discussed at the final analysis stage. In cases of missing data, the missing data mechanism will be explored, and multiple imputation may be applied as a sensitivity analysis as appropriate. However, from the pilot study, a high missing data rate it is not expected.

#### Plans to give access to the full protocol, participant level-data and statistical code {31c}

Royal Papworth Hospital NHS Foundation Trust, as sponsor and lead site, will manage participant level-data and de-identifiable data can be made available if requested by a member of the public. The statistical analysis code will be stored according to local standard operating procedures and can also be made available upon request.

### Oversight and monitoring

#### Composition of the coordinating centre and Trial Steering Committee {5d}

A Trial Steering Committee will be led by an independent chair. Per NIHR HTA guidelines, the TSC will be composed of an independent statistician, health economist and clinician, plus a patient representative and observers. The TSC will meet annually (or more frequently if necessary). An International Steering Committee will be similarly convened.

#### Composition of the data monitoring committee, its role and reporting structure {21a}

A Data Monitoring and Ethics Committee will be led by an independent chair who is an expert in the field. Per NIHR HTA guidelines, the DMEC will be composed of an independent expert statistician and clinician. Annual DMEC meetings will review progress against the agreed milestones, recruitment and safety. The trial statistician will provide the interim reports for the DMEC.

#### Adverse event reporting and harms {22}

**Fig. 3:**
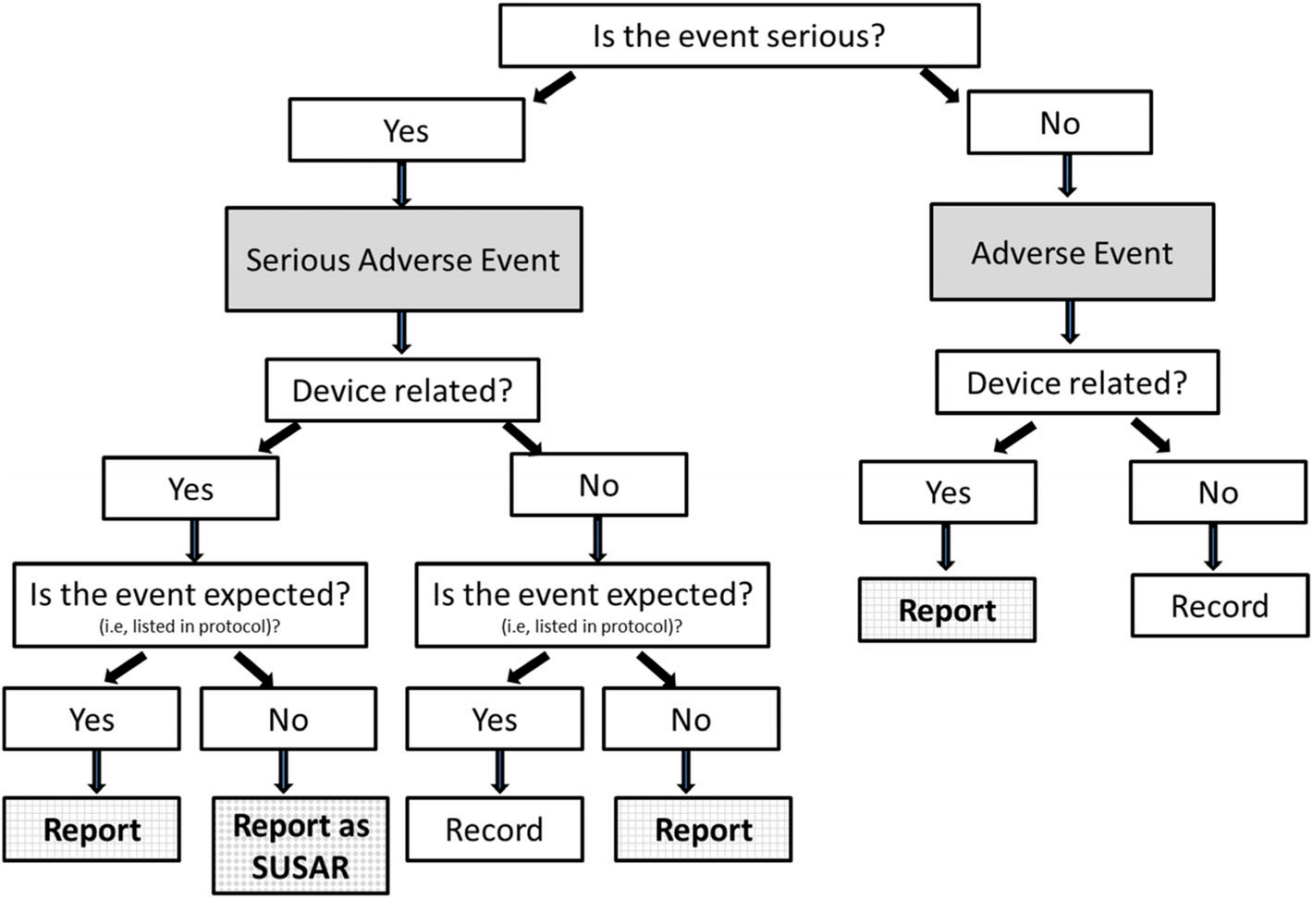
AE and SAE flow diagram

In cardiac surgery, postoperative complications are common. Only device-related adverse events (i.e., adverse reactions) and serious adverse events (SAE) that are device-related and/or ‘unexpected’ will be reportable to the sponsor. Please refer to Fig. 3.

Adverse events and SAEs will be collected from the time of tracheal extubation to discharge. From discharge up to 90 days after surgery, only SAEs will be collected. For events collected from tracheal extubation to discharge, the local PI will conduct medical assessment including the causality of the event. The sponsor also delegates responsibility for ‘expectedness’ assessment to the local PI. Unexpected events are those not listed in the study protocol.

All adverse events and serious adverse events will be coded using MedDRA version 23.1. Each event will be coded using the MedDRA Hierarchy with a corresponding Preferred Term, High Level Term, High Level Group Term and System Organ Class. All the expected AEs/SAEs were pre-coded where possible and reviewed by a medical professional. The AEs/SAEs will be coded as an ongoing process, with the coding staff communicating with the site or clinical staff as necessary for clarification.

#### Expected adverse events

Table 5 shows classified of adverse events that are ‘expected’ and should be recorded but not ‘reported’ unless causality is device-related.

**Table 5:** Table of expected adverse events

Event	Further details (where applicable)
**Acidosis**	
**Arrhythmias**	Including: -Supraventricular tachycardia or atrial fibrillation requiring treatment -VF/VT requiring intervention
**Aspiration of stomach contents**	
**Bleeding**	Requiring: -Transfusion -Return to theatre
**Escalation of respiratory support**	For example: (unplanned NIV/CPAP/re-intubation and invasive ventilation)
**GI complications**	Including: -Peptic ulcer/GI bleed/perforation -Pancreatic (amylase /1500iu) -Other (e.g. laparotomy, obstruction)
**Haemodynamic support**	Including use of: -Any inotropes -Intra-aortic balloon pump (IABP) -Need for invasive monitoring, e.g. pulmonary artery catheter -Vasodilator
**Infective complications**	Including: -Wound infection -Respiratory infection -Sepsis
**Low cardiac output state**	Requiring management with: -Swan-Ganz catheter -IABP -Left ventricular assist device
**Mediastinitis**	Including: -Requiring reoperation
**Neurological complications**	Including: -Stroke -Transient ischaemic attack (TIA)
**Pain in sternal wound/legs/arms incision sites**	
**Pneumothorax**	
**Pulmonary complications**	Including: -Re-intubation and ventilation -Tracheostomy -Initiation of mask CPAP ventilation after weaning from ventilation -ARDS
**Re-admission to ICU**	
**Renal complications**	Including: -New haemofiltration/dialysis -Acute kidney injury
**Resternotomy**	
**Thromboembolic complications**	Including: -Deep vein thrombosis -Pulmonary embolus
**Wound dehiscence requiring rewiring or treatment**	

#### Frequency and plans for auditing trial conduct {23}

Monitoring will be performed remotely for each site by the sponsor. In the event there are issues with the site in regards to documentation, consent or completion of data in OpenClinica then additional triggered on-site monitoring may be conducted at the discretion of the project management/QA team.

#### Plans for communicating important protocol amendments to relevant parties (e.g. trial participants, ethical committees) {25}

All study (including protocol) amendments will be submitted for approval to the relevant ethical and governance committees e.g. the REC and HRA in the UK. Sites will be informed of all approved minor or substantial amendments and will be asked to review and confirm approval at local site level. Participants will be informed and reconsented if deemed necessary by the sponsor. The study team will also provide protocol training for all protocol amendments. Monthly newsletters will also be published to ensure sites are informed of the latest study news.

#### Dissemination plans {31a}

Several members of the study team are national experts in clinical research and implementation of new research findings into clinical practice. Once completed and peer reviewed, social, professional and mainstream media will be contacted to inform as many people as possible about the study protocol, findings and recommendations for future clinical practice and health policy. It is expected that this multicentre trial will inform national guidelines for cardiac surgical, intensive care and anaesthesia and National Institute of Health and Care Excellence (NICE) clinical practice guidance. At the end of the study, feedback sessions and webinars will be arranged in order to present the results of the study to the Trial Steering Group and Investigators and Research Nurses from all recruiting centres. In addition, patients who participated in the study and service users will be informed about the final results of the study by newsletters, webinars, patient support groups and by presenting the results at research awareness days.

## Discussion

The study team is currently unsure about the implications the coronavirus pandemic will have on recruitment timelines. The study has experienced delays in trial commencement due to the pandemic and further operational issues in terms of site set-up and site recruitment are yet to be determined. The full effects will be discussed in the final paper.

## Trial status

Protocol version number: V3.0 dated 19 January 2022. This study is currently recruiting, with first patient being recruited on 7 October 2020. The planned end date of study is 31 January 2023.

## Abbreviations

AE: Adverse event
AKI: Acute kidney injury
ARDS: Acute respiratory distress syndrome
BiPAP: Bilevel positive airway pressure
BMI: Body mass index
CABG: Coronary artery bypass graft
CNS: Central nervous system
COPD: Chronic obstructive pulmonary disease
CPAP: Continuous positive airway pressure
CPB: Cardiopulmonary bypass
CRF: Case report forms
CRN: Clinical Research Network
CTU: Clinical trials unit
DAH: Days alive at home
DAH30: Days alive at home 30 days post-operative
DAH90: Days alive at home 90 days post-operative
DMEC: Data Monitoring and Ethics Committee
ECG: Electrocardiogram
eGFR: Estimated glomerular filtration rate
FiO_2_: Fraction of inspired oxygen
GP: General practitioner
HDU: High dependency unit
HFNT: High-flow nasal therapy
IABP: Intra-aortic balloon pump
ICER: Incremental cost-effectiveness ratio
ICH: International Council for Harmonisation
ICU: intensive care unit
ITT: Intention to treat
LOS: Length of stay
NICE: National Institute for Health and Clinical Excellence
NICOR: National Institute of Cardiovascular Outcomes Research
Physio: Physiotherapy
PI: Principle investigator
PPC: Postoperative pulmonary complications
PTUC: Papworth Trials Unit Collaboration
QALYS: Quality-adjusted life years
R&D: Research and development
RCT: Randomised controlled trial
REC: Research Ethics Committee
RR: Respiratory rate
sCR: Serum creatinine
SD: Standard deviation
SOT: Standard oxygen therapy
Sp02: Peripheral capillary oxygenation saturation
SUSAR: Serious unexpected adverse reaction
TIA: Transient ischaemic attack
TSC: Trial Steering Committee
VF: Ventricular fibrillation
VT: Ventricular tachycardia
